# A Superior Laxative

**Published:** 1909-10

**Authors:** 


A SUPERIOR LAXATIVE.—I have used Abbott's Saline Laxative and believe it to be superior to any laxative I have ever tried. The more I use it the better I like it. My patients say it is splendid, claiming that it does not gripe and does its work without any annoyance or bad feeling.—H. B. COBB, M. D., Perry, Michigan.

Samples of Abbott's Salines with complete literature may be obtained free on application to the Abbott Alkaloidal Company, Chicago, Ill.

